# Stone Pine (*Pinus pinea* L.) High-Added-Value Genetics: An Overview

**DOI:** 10.3390/genes15010084

**Published:** 2024-01-10

**Authors:** Ana Sofia B. Simões, Margarida Machado Borges, Liliana Grazina, João Nunes

**Affiliations:** 1 Association BLC3–Technology and Innovation Campus, Centre Bio R&D Unit, Rua Nossa Senhora da Conceição 2, Lagares da Beira, 3405-155 Oliveira do Hospital, Portugal; margarida.borges@blc3.pt (M.M.B.); liliana.grazina@blc3.pt (L.G.); joao.nunes@blc3.pt (J.N.); 2 BLC3 Evolution Lda, 3405-155 Oliveira do Hospital, Portugal

**Keywords:** disease resistance, drought tolerance, environmental stressors, functional genes, microsatellites, Pinaceae, conifers

## Abstract

Stone pine (*Pinus pinea* L.) has received limited attention in terms of genetic research. However, genomic techniques hold promise for decoding the stone pine genome and contributing to developing a more resilient bioeconomy. Retrotransposon and specific genetic markers are effective tools for determining population-specific genomic diversity. Studies on the transcriptome and proteome have identified differentially expressed genes PAS1, CLV1, ATAF1, and ACBF involved in shoot bud formation. The stone pine proteome shows variation among populations and shows the industrial potential of the enzyme pinosylvin. Microsatellite studies have revealed low levels of polymorphism and a unique genetic diversity in stone pine, which may contribute to its environmental adaptation. Transcriptomic and proteomic analyses uncover the genetic and molecular responses of stone pine to fungal infections and nematode infestations, elucidating the defense activation, gene regulation, and the potential role of terpenes in pathogen resistance. Transcriptomics associated with carbohydrate metabolism, dehydrins, and transcription factors show promise as targets for improving stone pine’s drought stress response and water retention capabilities. Stone pine presents itself as an important model tree for studying climate change adaptation due to its characteristics. While knowledge gaps exist, stone pine’s genetic resources hold significant potential, and ongoing advancements in techniques offer prospects for future exploration.

## 1. Introduction

### P. pinea L. (Stone Pine) as a Species and Its Ecological Significance

Pines trees are an integral part of European forests. These species play an important role in the actual context of global climate changes. The prevalence of different *Pinus* species depends on the climatic and geographic conditions. For example, *Pinus nigra* Arn. and *Pinus sylvestris* L. or *Pinus heldreichii* H. Christ, *Pinus uncinata* Ram, and *Pinus cembra* L. are predominant species in high and very high mountains, respectively, while *Pinus halepensis* Mill., *Pinus brutia* Ten., *P. pinea* L., *Pinus pinaster* Ait., and *Pinus canariensis* C. Sm. are endemic to the regions of the Mediterranean Basin [1]. Among the pine species native to this area, *P. pinea*, also known as stone pine, is one of the most appreciated for its aesthetic, ecological, culinary, and commercial uses. Although many authors dispute its autochthonous status, stone pine is mostly found in countries such as Spain, Italy, Portugal, Tunisia, and Morocco [2,3,4]. In Portugal, its populations have been increasing through reforestation efforts, mainly related to its resistance to pathogens such as *Bursaphelencus xylophilus*, the pine wood nematode.

The stone pine typically grows to a height of 10–25 m, although some exceptional specimens can reach up to 35 m. It has a broad, rounded canopy that resembles an open umbrella. The tree’s glabrous and greyish branches are often densely covered with dark green needles, typically arranged in pairs, and measuring around 10–15 cm in length. The needles persist on the tree for two or three years. It is a monoic species, although male and female flowers are formed in distinct structures. Pollen catkins are yellow in color and clustered at the base of the newly formed shoots. The large, distinctive cones grow to 8–14 cm in length at maturity. Each cone scale shelters two hard-shelled seeds (nuts) that are mostly dispersed through gravity [5].

Pine nuts, highly prized for their rich flavor and nutritional value [6,7], are utilized in various culinary dishes and are considered a delicacy [8]. According to industry reports from 2020, the pine nuts market was expected to expand by USD 332.23 million from 2020 to 2024, with a forecasted CAGR (Compound Annual Growth Rate) of 8%, including stone pine nuts among other pine nuts [9]. Industry reports from 2023 predict the growth of the Pine Nuts Market at a CAGR of 5.12% between 2022 and 2032 due to lower yields this season [10], with an anticipated increase of USD 712.34 million in market size [11]. The pine nuts market is growing due to rising consumer preference for healthy and natural foods, with a notable trend being their incorporation into plant-based and vegan diets. Regardless, supply fluctuations due to environmental factors and the labor-intensive harvesting process, contributing to higher costs and potential affordability limitations, are hindering the growth of pine nuts market [11].

In addition to its culinary significance, the stone pine has been valued for centuries for its timber [5], which is highly durable and resistant to decay. This is the second-most commercialized stone pine product, surpassed only by pine nuts. For example, in Northern Italy, timber production shows an average increase of 3 to 4 m^3^/ha per year and, under favorable ecological conditions, can reach 7 to 8 m^3^/ha per year [12]. The wood of the stone pine has been used in construction, furniture making, and boat building. Its unique grain pattern and warm color make it a sought-after material in woodworking. Furthermore, the stone pine is an important tree in its native ecosystems. The trees work as carbon sinks, which can be useful in climate change mitigation efforts using carbon reservoirs [13]. Its dense foliage provides shade and shelter for a variety of wildlife, and its extensive root system helps prevent soil erosion. The tree is well adapted to the Mediterranean climate, characterized by hot, dry summers and mild, rainy winters. It has a remarkable ability to withstand drought conditions and thrive in sandy and rocky soils [14].

Besides pine nut and timber production, stone pine has other economically valuable products. It is a source of resin, alternatively to *P. pinaster*. Its bark can be used industrially for tannin extraction. Lastly, pine nut shells and empty pine cones can be used as fuel, namely in pyrolysis [5]. The latter can yield a better short-term income than timber harvesting after long rotations [7].

Stone pine forests produce a significant amount of biomass in the form of needles, branches, tree trunks, cones, and nut shells [15]. These biomass resources can be utilized efficiently; for example, the needles and branches can be used for mulching, composting, or as a source of organic matter for bioenergy production through processes like pyrolysis or anaerobic digestion. The tree trunks can be processed into timber for construction, furniture, or other value-added wood products [8]. The tree is known for its highly valued edible pine nuts, which are considered Non-Timber Forest Products (NTFPs). The sustainable harvesting and processing of pine nuts can be integrated into a circular value chain. For instance, the shells and byproducts from pine nut processing can be utilized for bioenergy production or converted into secondary products such as animal feed or natural-fiber-based materials [16]. Stone pine forests play a crucial role in carbon sequestration by mitigating climate change impacts. Timber can be used in construction or as a substitute for high-carbon materials, contributing to a low-carbon circular economy. These forests can be managed sustainably, following circular bioeconomy principles. This involves adopting practices that prioritize biodiversity conservation, soil protection, and maintaining the health and productivity of the forest ecosystem. Sustainable forest management ensures the regeneration and renewal of stone pine forests, supporting the long-term viability of the circular bioeconomy [17].

Complementary to the direct monetary value generated by stone pine and its by-products, the ecosystem and provisioning services it provides also generate great economic impact. Cone scales and nut shells can be used in gardening. The presence of this species improves water retention and soil conservation, as well as weed control. The dust resulting from cone processing can be used in soil amendment approaches. Additionally, the umbrella-shaped crowns provide sun protection to the landscape and increase its aesthetic value [18].

Genetics plays a significant role in shaping the characteristics, adaptability, and overall health of wild populations of stone pine. Genetic diversity refers to the variety of genetic characteristics within a population. It is crucial for the long-term survival and adaptation of a species. In wild populations of *P. pinea*, genetic diversity provides the foundation for withstanding environmental challenges, such as diseases, pests, and changes in climate. Higher genetic diversity increases the chances of some individuals possessing traits that are advantageous for survival and reproduction [3]. Simultaneously, gene flow, the movement of genetic material (genes) between different populations, occurs through the dispersal of pollen and seeds. Gene flow can have both positive and negative effects on wild populations of stone pine. On the one hand, gene flow can introduce new genetic diversity into a population, potentially increasing its adaptability and resilience. On the other hand, excessive gene flow between distinct populations may lead to genetic homogeneity and loss of unique local adaptations. Another factor that modulates the genetic diversity of stone pine is inbreeding. Inbreeding occurs when individuals with closely related genetic backgrounds mate, leading to a reduction in genetic diversity. Genetic bottlenecks, which can occur due to natural disasters or human activities, result in a significant reduction in population size and genetic diversity. Despite its ability to adapt to everchanging environments (discussed further in Section 6), low levels of genetic diversity also put stone pine at risk for extreme climatic events, as its phenotypic plasticity only allows for slow local adaptation [19]. Understanding the genetic structure and diversity of wild populations of *P. pinea* is crucial for their conservation and management. Genetic studies can help identify genetically unique populations, prioritize conservation efforts, and design strategies to preserve or restore genetic diversity. Techniques such as genetic mapping, DNA fingerprinting, and marker-assisted selection can assist in assessing and managing genetic diversity in these populations. Overall, genetics plays a vital role in shaping the characteristics, adaptability, and long-term viability of wild populations of stone pine. Maintaining and enhancing genetic diversity, managing gene flow, and addressing the risks of inbreeding and genetic bottlenecks are crucial considerations in the conservation and sustainable management of this species in its natural habitats [20].

## 2. Scope of the Review and Methodology

**Figure 1:**
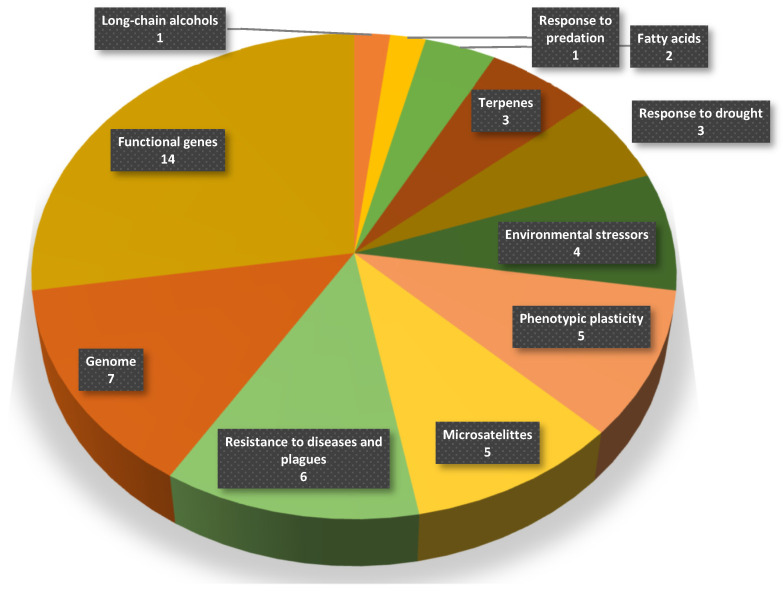
Results of the bibliographic research divided by theme and frequency of occurrence. The graph represents a total of 51 references.

The present work intends to compile and summarize relevant available information on stone pine genetics. Providing a concise review on the genetic components of this species will ensure that future works have a baseline to understand the underpinnings and influence of the genome on the most economic and ecologically important characteristics. Information regarding the genetics of this species is scattered and lacks comprehensive and systematic study. In this review, we used the platform Scopus (http://www.Scopus.com, accessed on 16 May 2023) to identify articles of interest. As a way to find relevant sources for this study, we limited the search to the targeted species (stone pine, or *P. pinea*) and to a 30-year period (1993 to 2023). To do this, we used the search query “TITLE-ABS-KEY (*pinus* AND *pinea*) SUBJAREA (bioc) AND PUBYEAR > 1992 AND PUBYEAR < 2024”. This query managed to find 200 documents. From those, only studies regarding genetics and hereditability were selected. This resulted in finding 51 articles regarding stone pine genetics, illustrated in Figure 1.

## 3. Genetic Diversity of the Stone Pine Species

### 3.1. Stone Pine Genome

**Figure 2:**
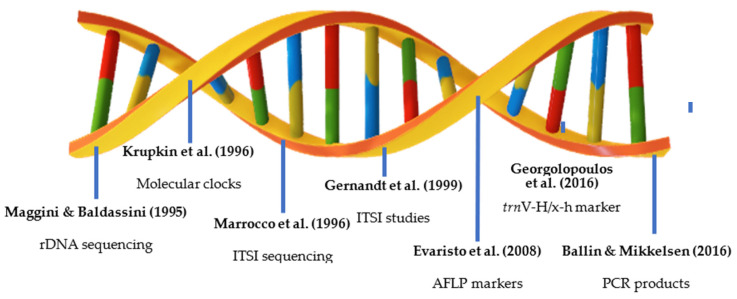
Chronology of studies on Stone pine’s genome [21,22,23,24,25,26,27].

Stone pine’s full genome is yet to be fully uncoded. Furthermore, its genetic information is severely understudied when compared with other members of the Pinaceae family. To study the genome of stone pine, several genomic techniques and approaches can be employed such as genome sequencing, de novo assembly, comparative genomics (comparing *P. pinea*’s genome with genomes of other plant species), functional and genetic mapping, and Quantitative Trait Locus (QTL) analysis. Figure 2 summarizes the chronology of works developed on Stone pine’s genome.

Stone pine genome studies started in 1995 with a paper on phage cloning, plasmid subcloning, and partial nucleotide sequencing of the *P. pinea* rDNA (ribosomal DNA) by Maggini and Baldassini (1995) [21]. It showed that the ribosomal band patterns observed are clearly characteristic of the species. This first attempt to study stone pine genetics was of great importance as it provided a way to genetically prove the phylogenetic relationships of this species with closely related taxa. Not long after, Krupkin et al. (1996) [22] showed that Mediterranean species were closely allied with members of sect. Pinea, which appeared to be polyphyletic. Krupkin et al. (1996) [22] worked with molecular clocks calibrated using two hard pine fossil observations, which introduced the integration of molecular clocks and fossil records in phylogenetics studies for this species. Marrocco et al. (1996) [23] reported the sequencing of the nucleotide sequence of the first internal transcribed spacer (ITSI) belonging to different ribosomal RNA genes from *P. pinea*. The nucleotide comparison of these regions did not show an appreciable sequence homology, which indicated that previous approaches were more effective for species identification. Gernandt et al. (1999) [24] worked with the nucleotide sequence from the ITS region of stone pine from Marrocco et al. (1996) [23]. This allowed for a comparative study of the same region from different species of the Pinaceae family. These species belong to the *Larix* and *Pseudotsuga* genera. This study identified dissimilarities of the obtained sequences when compared to the stone pine sequence. This analysis brought to light the phylogenetic relationships of the three genera within the Pinaceae family.

There was a 9-year period where no works were published on *P. pinea*’s genome, revealing a lack of interest in this particular subject. Retrotransposon markers were used by Evaristo et al. (2008) [25] to generate taxonomic data that are more consistent with morphological criteria than amplified fragment length polymorphism (AFLP)-based markers for *P. pinea*. Retrotransposon markers were more effective in determining population-specific genomic diversity than previous approaches based on polymorphic regions. Eight years later, Georgolopoulos et al. (2016) [26] reported a genetic marker (*trn*V-H/x-h) that was able to provide concrete phylogenetic and discriminatory information in the genus *Pinus*. The use of this primer resulted in the correct phylogenetic identification of 95 *Pinus* species, subspecies, and varieties with a high degree of posterior probability (0.99). This can be useful for pine identification even in contexts where DNA is degraded, such as in timber tracing, forensic botany, and palaeobotanical investigations. In the same year, Ballin and Mikkelsen (2016) [27] used principal component analysis (PCA) of high-resolution melting curves from PCR amplicons to cluster pine species from reference material, and to identify *P. pinea* from reference material. Despite the difference in approach, this work was effective in identifying stone pine samples.

### 3.2. Functional Genes

Studying functional genes in stone pine involves investigating the expression, regulation, and function of specific genes within the genome. This can be performed through transcriptomics, gene expression analysis through quantitative real-time polymerase chain reaction (qRT-PCR), proteomics, and genomic database mining. It is important to note that some of these techniques may require prior availability of genomic information or genome sequencing data for the targeted species. As the field of genomics advances, new techniques and approaches are continually being developed and could be used to further understand the functional genes and their roles in the biology of stone pine.

So far, it was possible to identify and characterize ten stone pine genes, related to adventitious meristem formation, pathogen attack response, response to atmospheric CO_2_, and water stress (Table 1). Simultaneously, some authors have tried different approaches to study the transcriptome and proteome of this species.

Genome-wide profiling (transcriptomics, proteomics, and metabolomics) provides unprecedented opportunities to understand the complexity of coordinated gene expression in trees. Despite the developments in this area, the works regarding stone pine transcriptomics and proteomics are scarce and temporally distant.

Regarding the transcriptome of stone pine, two papers attempted to characterize the transient gene expression by quantifying specific transcripts during two growth stages: adventitious shoot bud formation [33] and embryogenesis [34]. Quantitative real-time PCR analysis was performed to confirm the differential expression of 30 candidate genes regarding adventitious shoot bud formation. Many genes were found to have a homology with known sequences; however, only a few were related to functional genes. Expression profiling with microarrays suggested that a large number of genes were involved, making it impossible to pinpoint a single gene responsible for this process. The most expressed genes were homologous with PAS1, CLV1, the SNF2 domain, ATAF1, and ACBF, genes involved in developmental regulation [33]. The molecular regulation from early to late embryogenesis was reviewed by Trontin et al. (2016) [34], through an analysis of known “omics” from other conifers. Their results show that embryogenesis may mainly arise from the spatiotemporal modulation of auxin-, gibberellin-, and abscisic acid-mediated responses. Additionally, they showed that several important processes are apparently conserved in plants, in particular the early organization of apical–basal embryo patterning during late embryogenesis.

The stone pine proteome has been explored in three different articles, mainly involved in developing tools to use a proteomic approach to phylogenetics. In 2004, Alvarez et al. (2004) [35] spearheaded this technique by analyzing the globulins present in the megagametophyte. They observed that they present a high degree of variation, meaning that the evaluated populations were highly polymorphic for these proteins. Loewe et al. (2018) [36] reported that the protein profile analysis based on SDS-PAGE 1-D and 2-DE allowed a clear differentiation among Chilean stone pine macrozones. Whether this differentiation has a genetic or environmental control remains unclear. Amaral et al. (2021) [37] evaluated the dynamics of the needle proteome of stone pine upon *Fusarium annosnatum* (a fungal pathogen) inoculation and found that the regulation of gene expression through epigenetic mechanisms may support the pine’s response to infection (further discussed in the section “Genetic responses of stone pine to external factors”).

Isoenzymes have been used in some studies to try to establish phylogenetic parallels to DNA analyses. These enzymes were specifically targeted due to their conservation through evolution. Studies on isoenzymes were pioneered by Fallour et al. (1997) [38]. This work reported that isoenzymes isolated from megagametophytes and seeds were able to show differences between 17 stone pine populations in the Mediterranean Basin. The results also showed that interpopulation differences were greater than intrapopulation differences. This means that these enzymes are good molecular targets for selecting good breeding populations. Gad et al. (2012) [39] also worked with isoenzymes, this time to ascertain the use of these enzymatic markers in phylogenetics. The authors were able to distinguish three Tunisian pine species and correctly cluster them to reflect their evolutionary relationships. This proves conclusively the usability of isoenzymes as profiling tools for taxonomy.

The knowledge regarding the genetic information of expressed enzymes from stone pine is very limited. Stermitz et al. reported in 1994 [40] that the piperidine alkaloid profile of pine trees can be determined by genetic variation and the specific conformation of these compounds can interpolate enzyme expression. The dominance of a specific form of piperidine alkaloid in stone pine points to the presence of imine reductase activity and chemically isolates the species from other pines such as *Pinus edulis*, *Pinus fexilis*, *Pinus jeffreyi*, *P. nigra*, *Pinus ponderosa*, and *P. sylvestris*. Wolff et al. (1997) [41] detected the presence of the enzyme Δ5-desaturase, characteristic of Gymnosperms, in samples from 49 pine species, including stone pine. Pines that are restricted to warm-temperate regions have a low Δ5-desaturase activity that results in a low total content of Δ5-olefinic acids. A decrease in Δ5-desaturase activity can be considered the result of an evolutionary mechanism that isolated stone pine to these regions.

Regarding enzymes present in seeds, Tommasi et al. (1999) [42] studied stone pine seeds as a proteome model for orthodox seeds (seeds that survive dry conservation ex situ). This approach found that stone pine seeds have dehydroascorbate-reducing proteins (DHA-reducing proteins) but did not have ascorbate peroxidase, as was the case of species with recalcitrant seeds. The team postulated that orthodox seeds do not need ascorbate peroxidase, as they are dry with low oxidative metabolic activity and, therefore, low hydrogen peroxide production. Gonzáles-Andrés et al. (1999) [43] also worked with seeds. However, the main goal of this work was to establish if isoenzyme expression profiles could be used to distinguish between pine species autochthonous to the Iberian Peninsula and Canary Islands. The identification of stone pine as an isolated species was successful, which did not occur for all tested pines. Stone pine’s isoenzymatic profile was constituted by glutamate oxalacetate transaminase (shared with *Pinus cannariensis*), esterases (unique for each species), acid phosphatases, and superoxide dismutase. In 2000, a study by Ranaldi et al. [44] reported that the enzyme isocitrate lyase can be inhibited by phosphate. This conclusively proved the existence of this active enzyme in stone pine. Then, 19 years later, Faraoni et al. (2019) [45] studied enzymatic profiles during seed germination. The main focus of this study was the seed germination response to altered gravitational conditions and, although this is not relevant to the current review, this work highlighted several enzyme groups present in stone pine nuts. The authors were able to monitor the expression of six different enzymes: isocitrate lyase, malate synthase, glyoxylate cycle, 3-hydroxyacyl-CoA dehydrogenase, isocitrate dehydrogenase, pyruvate kinase, and glucose 6 phosphate dehydrogenase. The detection of these enzymes conclusively proved their involvement in the germination process.

Hu et al. (2022) [46] successfully engineered *Escherichia coli* to convert the lignin-derived monomer cinnamic acid into pinosylvin by introducing an enzyme from stone pine. This enzyme, stilbene synthase (PpSTS), was efficient in the biosynthesis of pinosylvin and demonstrated the potential application for the biosynthesis of products derived from cinnamic acid. This work is one of the few examples of the direct use of stone pine genetics in industrial applications.

## 4. Genetic Responses to External Factors

### 4.1. Microsatellites

Microsatellites, also known as simple sequence repeats (SSRs), are short repeated DNA sequences scattered throughout the genome. Studying microsatellites in stone pine can provide valuable information about the genetic diversity, population structure, evolutionary history, and breeding strategies of this species. They are mainly used in quantitative genetic studies from stone pine. Their importance seems to be highlighted by the investment in this subject, proving to be the most studied among these papers found for this species. Table 2 summarizes the findings from stone pine’s microsatellite studies.

The first reference to stone pine microsatellites was by González-Martínez et al. in 2004 [47]. In this work, it was found that no trans-specific microsatellite was polymorphic for the species and, consequently, the de novo development of microsatellites seems mandatory. Vendramin et al. (2008) [48] proposed that this lack of polymorphism is not necessarily a negative trait in the study of these DNA sequences. As genetic variation is generally considered a prerequisite for the adaptation to new environmental conditions and stone pine shows such low levels of polymorphism, it appears to have passed through a severe and prolonged demographic bottleneck. This bottleneck was followed by the subsequent natural- and human-mediated dispersal across the Mediterranean Basin, resulting in an abundant and widespread plant species with little genetic diversity at both chloroplast and nuclear markers. This is unreported for any other species with such a great demographic distribution. In concordance with the previous findings of Vendramin et al. (2008) [48], Soto et al. (2010) [49] observed that genetic diversity differences across species in the *Pinus* genus were not consistent with general predictions relating distribution range and intraspecific variation. Thermophile species, such as the stone pine, suffered further habitat fragmentation and intense range contractions into smaller coastal refugia (supported by the Iberian fossil record). With the forecasted rapid global warming in Europe, especially in the Iberian Peninsula and other Mediterranean mountainous regions, this lack of polymorphism may prove to be an advantage for the adaptation of this species. Pinzauti et al. (2012) [50] isolated 12 nuclear microsatellites from genomic and cDNA sequences. These microsatellites along with the cDNA sequences showed, as in previous works, low polymorphism. Jaramillo-Correa et al. (2020) [19], despite being the latest attempt to use microsatellites, found similar results to those of the previously mentioned author. The patterns of nucleotide diversity, molecular adaptation, and genetic load across 177 gene-*loci* demonstrated that the widespread and outbreeding stone pine has unprecedentedly low genome-wide levels of genetic diversity.

### 4.2. Resistance to Diseases and Plagues

Determining genetic responses to pathogens in stone pine, whether it pertains to diseases (such as fungal infections) or plagues (such as nematodes and insects), involves molecular, genetic, and genomic approaches. Common methods include pathogen inoculation and the evaluation of disease symptoms, quantitative genetic analysis, genetic mapping and QTL analysis, transcriptomics, and gene expression profiling. These techniques help uncover the genetic components and mechanisms of resistance, aiding in breeding and disease management efforts for enhanced pathogen resistance in stone pine.

The diseases that affect pine tree can be of viral, bacterial, or fungal nature. So far, we have only started to understand the machinery behind stone pine’s response to fungal infections, and even then, the results are very preliminary. Three species of fungal pathogens have been studied in the scope of stone pine–fungal pathogen interactions: *Heterobasidion annosum*, *Heterobasidion irregulare*, and *Fusarium circinatum*.

The genus *Heterobasidion* causes root and butt rot in pines as well as broadleaved species [32]. Seeing as this fungal disease has a broad range of hosts it can infect, understanding resistance mechanisms prove to be important to control its devastating actions.

*F. circinatum* infection is the main cause of pine pitch canker. Seedling mortality increases and well-established trees suffer die back, stem cankers, and branch and trunk girdling, eventually leading to death. The effectiveness of this pathogen has led to its status as the most important conifer pathogen on a global scale [51].

**Figure 3:**
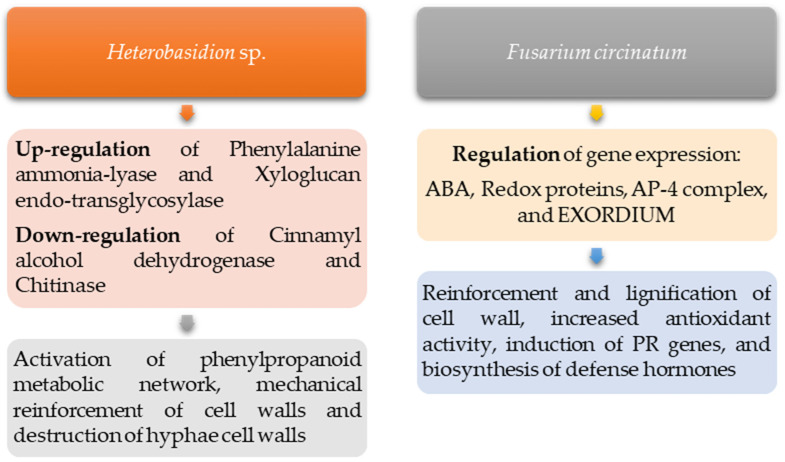
Disease resistance mechanisms identified for stone pine.

Figure 3 illustrates the disease resistance mechanisms found in stone pine for the three mentioned pathogens.

Pepori et al. (2018) [32] reported that several defense-related genes and terpenes were highly induced after infection by the two different *Heterobasidion* species (a common phytoparasitic fungal genus). An up-regulation was detected for phenylalanine ammonia-lyase (the initial key step in the phenylpropanoid metabolic network) and xyloglucan endo-transglycosylase (involved in the mechanical reinforcement of cell walls under pathogen attack). Alternatively, cinnamyl alcohol dehydrogenase (important for lignin biosynthesis) and chitinase (involved in the destruction of hyphae cell walls) showed a down-regulation for both pathogens. The up-regulation of transcripts as well as the accumulation of terpenoids revealed that a systemic signal is inducible in stone pine by both pathogens.

Seeing as the stone pine seems to be naturally resistant to *F. circinatum* infection, it becomes important to study this plant–pathogen interaction as it can help determine why other species are susceptible and modulate antifungal solutions. After *F. circinatum* infection, Zamora-Ballesteros et al. (2021) [51] studied the transcriptome stone pine and revealed an early perception of the pathogen with a coordinated defense activation through the reinforcement and lignification of the cell wall, the antioxidant activity, the induction of PR genes, and the biosynthesis of defense hormones. Amaral et al. (2021) [37] evaluated the dynamics of the stone pine needle proteome upon *F. circinatum* inoculation by GeLC-MS/MS and found a crosstalk between abscisic acid (ABA) and the regulation of gene expression through epigenetic mechanisms. Chloroplast redox proteins may allow redox homeostasis to be maintained, as well as non-enzymatic antioxidants such as anthocyanins, flavonoids, and vitamin B6, which constitutes an additional resistance mechanism. Important cell proteins (AP-4 complex and EXORDIUM) control membrane trafficking under pathogen infection and assist in the plant’s defense.

**Figure 4:**
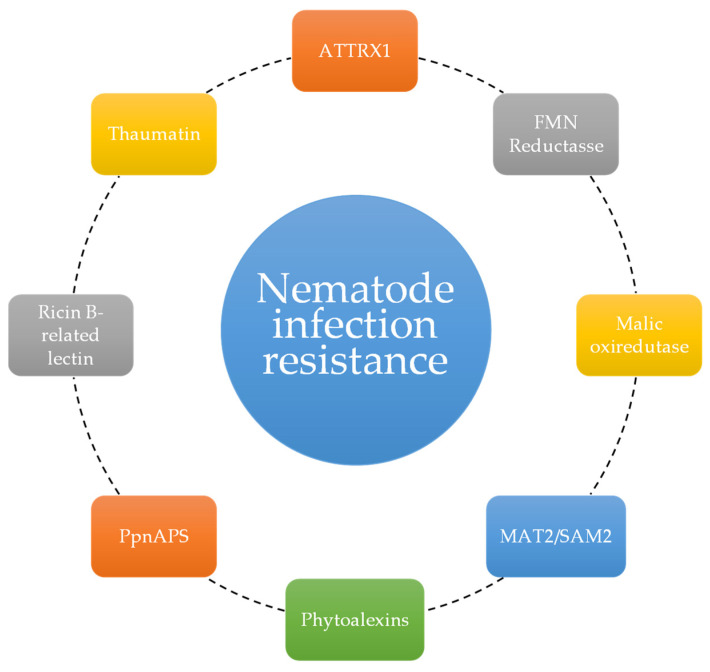
Enzymes and proteins involved in stone pine’s *B. xylophilus* infection resistance.

The genetic mechanisms involved in pathogen resistance in stone pine have been studied by several authors mainly regarding its natural resistance to the pinewood nematode, *B. xylophilus*, the organism responsible for pine wilt disease. The nematode uses an entomological vector, the longhorn beetle *Monochamus galloprovincialis*, to infect its hosts [30]. The nematode then invades the resin canals of the xylem and cortex, where it feeds on epithelial cells. This causes blockage of the vascular function and cavitation, and disrupts water transport. The main symptoms of pinewood nematode infection are a lack of resin exudation, lowered water potential, needle discoloration or browning, and, ultimately, death [52]. This nematode is extremely nefarious to other pine species and can be economically devastating. Thus, the undercovering of resistance processes can help in finding nature-based solutions for other, less resilient tree species. Figure 4 highlights the enzymes and proteins involved in the pathogen resistance pathways.

In 2011, Franco et al. [30] pinpointed the expression of Pathogenesis-related proteins 4 after inoculation with the pinewood nematode. The expression of proteins is associated with pathogen attack response mechanisms encoded in the genes *ATTRX1* and *MAT2/SAM2*. These proteins are mainly associated with osmotic stress, oxireductive processes, and cell death, demonstrating that these processes may be integrated in the tree’s ability to survive nematode infection. The comparative transcriptome analysis by Santos et al. (2012) [52] showed that stone pines infested with the pinewood nematode highly expressed ricin B-related lectin, FMN reductase, and malic oxidoreductase. Their differential expression is a common mechanism in the general defense against a multitude of plant pathogens. Phytoalexins, that show nematocidal activity, were also differentially expressed after inoculation. The pinewood-nematode-related thaumatin, a disease resistance protein, was also detected in this work. This protein is especially interesting as it is required at the transcriptional level in the reaction to stress responses and environmental changes. To understand the role of terpenes in plant–nematode interactions, Trindade et al. (2016) [31] studied the α-pinene synthase gene expression in in vitro axenic shoot cultures of stone pine. The semi-quantitative PCRs revealed that after infection, there was a clear response by the tree, corresponding to an increased expression of *Pinus pinea* α-pinene synthase (PpnAPS). Despite the promising result, it was not possible to determine to what extent the differences in gene expression level are related to pathogen resistance.

### 4.3. Environmental Stressors

The genetic components of the response to environmental stressors in stone pine can be determined by a multidisciplinary approach that combines molecular biology, genetics, genomics, and physiological analyses. This knowledge can be used to develop breeding programs, genetic improvement strategies, and management practices to enhance stress tolerance and the adaptability of stone pine populations. For this species, we have only started to uncover the genetic components of the response to increases in UV-B radiation and atmospheric CO_2_ responses and to water stress. Table 3 summarizes the information of stone pine’s response to environmental stressors.

Petropoulou et al. (1995) [53] studied the photosynthetic response of plants exposed to increasing UV-B radiation. The results suggest that plants are negatively affected by radiation. UV-B radiation is considered a potential oxidative stress and may induce the expression and increased activities of anti-oxidative enzymes like superoxide dismutase.

The way organisms react to environmental factors, including atmospheric CO_2_, depends on multiple factors and few of them can be related to genetic traits. *AOX* genes were isolated by Frederico et al. (2009) [28] using a PCR approach and have been suggested to be involved in plant reactions upon increasing CO_2_ contents in the atmosphere. The definitive way in which they modulate this response is yet to be determined. However, this result seems promising regarding climate change adaptation.

Regarding water stress, Perdiguero et al. (2013) [54] found promising candidate genes for the drought stress response. These include genes related to carbohydrate metabolism, including glycosyltransferases or galactosidases, sugar transporters, dehydrins, and transcription factors. They can be classified into functional categories such as metabolism, cell rescue and defense, and transport- and transcription-related genes. Stone pine appears to be a more sensitive species in this regard as it displays a fast and strong transcriptional response. This investigation was continued in Perdiguero et al. (2015) [55], with a special focus on dehydrins. Amongst the identified dehydrins, the transcript level of K2-dehydrins increased significantly under drought stress. A higher accumulation of these in aerial parts of the plant could underlie the higher tolerance to drought for this species. The fact that this increase takes place in the aerial parts of the tree could indicate a specific functionality in these organs during water stress signaling, which could allow a good retention of water under drought.

## 5. Chemical Profiling and Phylogenetics

### 5.1. Terpenes

Terpenes are common constituents of essential oils. Stone pine is rich in essential oils and their characterization has been the target of several studies. These compounds are responsible for the characteristic aroma and resinous scent associated with the species. Different pine species often have distinct terpene profiles, providing a chemical fingerprint of the species and its variations. They can assist in distinguishing stone pine from other pine species and subspecies. Additionally, terpene analysis can provide insights into the genetic and environmental factors that influence terpene production in pine populations. Table 4 contains the terpene profiles, and respective chemotype designation, identified for stone pine.

Roussis et al. (1995) [56] studied foliage terpene profiles of five pine species (*P. brutia*, *P. pinea*, *P. nigra*, *P. cannariensis*, and *P. halepensis*). Each of the five species represented its own recognizable chemotype, therefore allowing the identification of stone pine from unknown samples. Although the foliage chemical constituents allowed for the identification of characteristic chemotypes, they fail to reproduce evolutionary relationships. Da Silva et al. (2001) [57] took a similar approach and attempted to perform a chemotaxonomic identification of ten pine species. This study targeted monoterpenes, namely differences in enantiomers between the tested species. Of those, stone pine was easily distinguished from other pines due to its unique limonene profile. This way, it is possible to conclusively distinguish stone pine from other Pinaceae family members. Then, 20 years later, Gad et al. (2021) [58] revisited the hypothesis of terpene profiling for species identification. The results of this study were concurrent with the previous works, and stone pine could be isolated from other species based solely on its terpene chemical profile.

### 5.2. Fatty Acids

Examining the composition and distribution of fatty acids within living organisms can give valuable insights into evolutionary relationships, species identification, and understanding ecological roles. By combining fatty acid analysis with other taxonomic approaches, such as molecular biology and morphological studies, we can refine and improve the classification of organisms. Table 5 lists the fatty acids, from stone pine, that were used for chemotaxonomic studies.

In 1997, Wolff et al. [41] attempted to find taxonomic parallels between classical classification and fatty acid analysis for pine seed oils in 49 different pine species. All tested species (including stone) were correctly clustered in sects. Nasri et al. (2005) [59,60] determined the fatty acid contents of stone pine seeds from different forests in Tunisia. The results are consistent with those obtained for other *Pinus* species. These papers report that fatty acid contents did not vary between populations, suggesting that this species is fairly genetically homogenous in the country. Moreover, the authors postulate that this low genetic diversity may be due to the anthropic intervention on this tree’s distribution, where the same genotypes were introduced in several different locations.

### 5.3. Long-Chain Alcohols

Long-chain alcohols can be valuable chemical markers to differentiate pine species, assess genetic diversity and population structure, understand environmental adaptations, infer phylogenetic relationships, and detect hybridization and introgression events. The integration of morphological traits with chemical profiling provides a comprehensive approach to refine species classification and deepen the understanding of genetic and ecological aspects of stone pine.

Recently, Gaspar et al. (2023) [61] used foliar n-Alkane and long-chain alcohols profiling to determine their applicability in distinguishing pine species. Stone pine had a unique chemical profile that was successfully used in chemotaxonomic analysis. All the tested species had profiles that accurately reflected their phylogenetic relationships.

## 6. Hereditability, Evolution, and Adaptive Traits

### 6.1. Response to Drought

**Figure 5:**
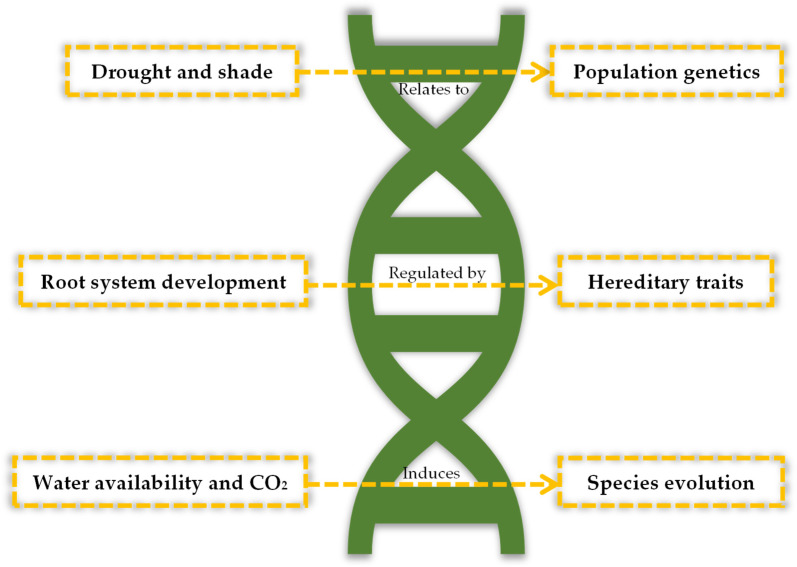
Drought-response-related parameters in stone pine and associated genetic processes (population genetics, hereditary traits, or species evolution).

Although information regarding the genetic components of drought resistance in stone pine are scarce, studies on the hereditability of associated traits can be helpful in understanding the relationship between genetics and phenotypic plasticity. Hereditary traits are inherently related to the species genome. Once the capacity to survive drought periods is determined to be inherited through generations, breeding programs can focus on selecting the best individuals to perpetuate this genomic-based trait. Figure 5 represents the drought-response-related parameters evaluated in the literature.

Pardos et al. (2018) [62] studied the response to different gradients of drought and shade on stone pine from different geographic origins within Spain. The results show that the response was similar for all individuals irrespective of their origin. This means that this trait, although genetically based, is transversal to all populations and therefore hereditary. Additionally, it was determined that all populations showed a phenotypic plasticity that enabled their adaptation to new environmental conditions at the local level. This plasticity ultimately resulted in differences in adaptive traits for the studied geographical origins. In the same year, Andivia et al. (2018) [63] reported that drought resistance is modulated by root system development. This way, this study focused on the link between root characteristics and phylogenetics. The authors found that stone pine and other Mediterranean species have a faster root growth and a stronger root system. This work proved that drought resistance is not only hereditary within Mediterranean pines, but also an evolutionary adaptation directly correlated to drought conditions. In 2023, Férriz et al. [64] tested the multidimensional functional trait variability in both *P. pinea* and *P. pinaster* in response to drought and CO_2_. The results showed that traits were more affected by the environmental conditions (water availability and CO_2_ concentration) than by interspecific differences. However, stone pine was more competitive under water stress, indicating an evolutionary discrepancy between both species.

### 6.2. Response to Predation

Predation is one of the main drivers for species evolution, alongside climatic factors. From seed predation to interactions with herbivores and pathogens, these selective pressures influence the genetic composition and adaptations of the species. As stone pine continues to coexist with its predators, adaptive traits will shape the species response to this evolutionary pressure. The best fitted phenotypes are undoubtedly perpetuated through hereditary traits.

Bogdziewicz et al. (2021) [65] determined the effect of seed predation on masting (irregular and periodically synchronous production of seeds in perennial plants) interannual variability and synchronicity in several Mediterranean tree species, including the stone pine. This paper reports that predation is made for the selection of stone pine individuals with interannual variability and reproductive synchronicity. This characteristic seems to be modulated within populations and therefore hereditary.

### 6.3. Phenotypic Plasticity

**Figure 6:**
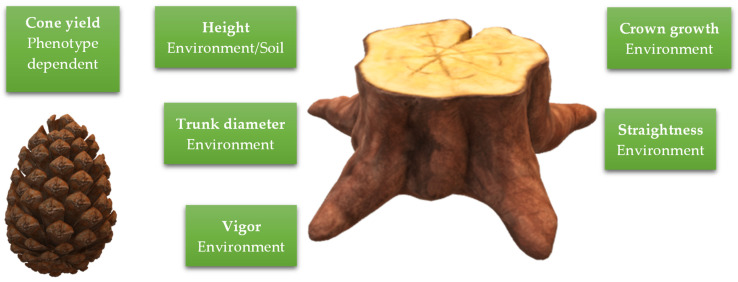
Characteristics involved in phenotypic plasticity in stone pine.

Phenotypic plasticity, the ability of a species to develop different observable traits in response to varying environmental cues and stresses, is one of the main drivers behind the use of stone pine in reforestation efforts and landscape design. Stone pine shows great phenotypic plasticity despite its low genetic diversity, making it a highly adaptable and resilient species. The use of this pine in forest management is rooted in the fact that most positive traits are developed due to physiological responses to external factors. Instead of selecting improved genotypes for breeding programs, decision-makers can choose target locations to ensure favorable outcomes for this species. Phenotypic plasticity can be observed in several different morphophysiological aspects of the tree, namely cone and seed yield, and growth traits. Figure 6 summarizes the characteristics evaluated in phenotypic plasticity studies.

Many authors have looked at cone production and seed yield as a marker for plasticity. In 2003, Mutke et al. [66] reported that when evaluating cone yield for a Spanish clonal bank with the intent of selecting candidate genotypes, the authors found that there were no notable differences between populations. Their results showed that cone production was not dependent on population and that for the same location, it was impossible to select an improved genotype. Two years later, Mutke et al. (2005) consolidated their study on cone yield and found that cone size and productivity had a low degree of genetic determination (17%). This, in turn, justifies their previous findings [66], where no differences were detected between genotype candidates for this characteristic.

Phenotypes can also be determined based on growth traits, evaluating parameters like shoot growth and frequency, trunk diameter, and crown growth. Carrasquinho et al. (2013) [67] found that when measuring the diameter at breast height for two different population sites (Sines and Tavira, Portugal), significant differences were observed. The authors proposed that the higher values found for populations in Sines were due to genetic variability even in the presence of the contradictory literature. Whether there is a genetic component behind the results or not, the main conclusion remains the same: different sites differ in trunk diameter. In the same year, Mutke et al. (2013) [68] worked with experimental plots established around a decade before in 40 provenances from Lebanon, Turkey, Greece, Italy, France, Spain, Portugal, Marocco, and Tunisia. The authors found that when targeting height as a growth parameter, samples were homogeneous among provenances but differed among sites. The results indicated that stone pine varied more due to soil conditions and characteristics than due to population genetics. Five years later, Loewe-Muñoz et al. (2018) [69] evaluated several parameters of tree growth (height, diameter, crown growth, and vigor and straightness) and determined that these were more affected by environmental factors (rainfall and temperature) than by tree spacing or seed origin.

### 6.4. Stone Pine and Other Related Species

Stone pine’s genetics has been the target of interest since 1967, when Brunori and D’Amato published a paper on the contents of nucleic acids of *P. pinea* seeds [70]. In comparison, the closely related *P. pinaster* only had its first work on genetic contents in 1986 [71]. However, the interest in stone pine genetics started early, and the effort level throughout the years in this research has been lower than for other pine species. When performing a search in Scopus using the same query string but changing “pinea” for “pinaster” and “radiata”, the results change from 200 documents (for stone pine) to 529 (for *P. pinaster*) and 521 (for *Pinus radiata*). The amount of available literature is more than double for closely related pine species than for stone pine.

Genome sequencing provides information on the complete genomic information of a certain organism. It not only highlights the coding sequences (functional genes) but also transcription factors, “junk” DNA, and other important components that make up the genome’s machinery. Knowing the full genome of a certain species can provide tools for species amelioration as well as highlight the genetic backgrounds for a certain trait or physiological process. Modeling and homology studies are extremely useful when genome-wide sequencing has not been carried out. However, these approaches are not enough to determine gene expression and function with high degrees of confidence [72]. Genome sequencing is still in its early stages for conifer species. In 2010, the full genome sequence for another pine, *Pinus taeda*, was published [73]. In 2011, a large database on the *P. pinaster* transcriptome was made available [74]. Stone pine’s genome sequencing is yet to be completed and most of its transcriptome remains a mystery.

Some conifers have also been the target of genome-wide association studies (GWASs), as is the case of *Pinus contorta* [75] and *Picea abies* [76]. A search for “*P. pinea*” in the National Center for Biotechnology Information platform (https://www.ncbi.nlm.nih.gov/, accessed on 25 July 2023) shows that 114 gene sequences have been published for stone pine. The same search for “*P. pinaster*”, “*P. radiata*”, and “*P. abies*” shows 4768, 1179, and 1424 gene sequences, respectively. These numbers reveal the investment in genetic research for these related species when compared with stone pine.

Regarding breeding programs, stone pine has been the target of some studies. For example, Mutke and Gil (2000) [77] proposed the establishment of highly productive stone pine forests in Castille-Léon, Spain, whereas Loewe-Muñoz et al. (2017) [78] proposed the establishment of pine nut products in central Chile. Both were solely based on the selection of phenotypes with high cone yields. A genetic basis for this selection would have been beneficial in order to choose, for instance, disease-resistant genotypes or more resilient trees. Examples of genetics-based programs can be found for *P. pinaster*, *Pinus flexilis*, and *P. radiata*. For *P. pinaster*, it was possible to find a breeding program based on improving height and stem straightness using genotype selection [79]. For *P. flexilis*, the authors suggested the use of exome sequencing to select disease-resistant plants [80]. Finally, for *P. radiata*, the acceleration of genetic gains has been proposed to genetically improve seed orchards [81].

In 2004, EUFORGEN (https://www.euforgen.org/, accessed on 25 July 2023) published guidelines on stone pine genetic conservation and use [5]. This was a great starting point for genotype valorization. However, no further efforts were made for stone pine. Other pines have been the target of such studies and efforts more recently (from 2014 to 2022), as is the case of *P. cembra* [82], *P. sylvestris* [83], and *P. contorta* [84].

## 7. Valorization of Genetic Diversity Markers

### 7.1. Genetic Markers

Stone pine genetic markers can conclusively differentiate between stone pine samples and other closely related taxa, as well as decode phylogenetic relationships between them. Several different genetic markers can be used in phylogenetic studies, namely standard markers (like the ITS region) or custom markers (like *trn*V-H/x-h designed in [26]). The ITS region is known for its use in DNA barcoding [85] and is, therefore, used in this type of analysis. The reviewed works [21,24] were able to taxonomically isolate stone pine using ITS sequence analysis with only one exception [23]. ITS sequence analysis requires a good DNA sample, with this specific nucleotide sequence intact. Any degradation that affects a portion of the sequence will invalidate the results. In cases where the DNA sample is degraded or difficult to obtain, it can be advantageous to use a more specific marker [26]. These markers are usually species- or genus-specific and may not be useful in phylogenetic assessments. However, their specificity allows for species identification from mixed or unknown samples.

### 7.2. Retrotransposons

Retrotransposons are mobile DNA sequences normally associated with viruses. Plant genomes are rich in retrotransposons. The diversity associated with these sequences in plants makes them a great candidate for genetics-based phylogeny studies. It was found that retrotransposons are excellent markers for taxonomic analysis, going as far as elucidating evolutionary relationships between plant species [86]. Stone pine retrotransposons provide taxonomic identifications more closely aligned with morphological data than other genome fragment analysis [25]. This fact illustrates the importance of these sequences for the validation of taxonomic identification. Additionally, given their reliability, it can be inferred that retrotransposons could be useful in sample validation for pine nut commercialization.

### 7.3. Restriction Enzymes

Generating DNA fragmentation profiles with restriction enzymes is a reliable and cost-effective technique for non-model plant species [87]. The fact that it can be applied to non-model species is of extreme importance as many genetic identification techniques rely on the comparison with genome sequence databases, which are created mainly for model species. This approach to the genetic validation of evolutionary relationships has shown great results, even for stone pine. It allowed for distinguishing stone pine samples from closely related species [22]. Restriction enzyme profiles provide support where other techniques fail, namely in understudied plants and fossil records.

### 7.4. Nuclear DNA

Nuclear DNA can provide information regarding intra- and interspecific genomic diversity, and the functional genes present in certain plants. Microsatellites, which are short tandem repeated DNA motifs, have been extensively studied as genetic diversity markers [88]. Microsatellites are evaluated regarding their polymorphism: high variability indicates genetic diversity while low variability indicates genetic uniformity. It is rare that microsatellites can be used for interspecific comparison, as their transferability is usually low [88]. In stone pine, microsatellites indicate low intraspecific polymorphism [19,47,50]. The low degree of genetic diversity within stone pine does not mean that its genome is simple. Stone pine’s nuclear DNA is extremely rich in environmental adaptation and disease-resistant-related genes. Regarding the latter, stone pine’s nuclear DNA is rich in pathogenesis-related genes. These genes explain the broad range of diseases and plagues that this pine is immune to [51].

### 7.5. Mitochondrial DNA

Mitochondrial DNA in plants is mainly responsible for encoding vital respiratory cycle enzymes. This type of DNA shows great plasticity due to consistent rearrangements and foreign DNA integration [89]. This makes mitochondrial DNA less reliable for phylogenetic studies than, for example, chloroplast DNA. Despite the unreliability, some studies have been able to report correct phylogenetic alignments using mitochondrial DNA, namely for stone pine [27]. This shows that even sequences with high degrees of plasticity can translate to invaluable evolutionary genetic information.

### 7.6. Chloroplast DNA

Chloroplast genomes are smaller than nuclear genomes and, unlike mitochondrial DNA, their nucleotide sequences are stable over time [89]. The smaller size makes cpDNA a better candidate for sequencing in phylogenetic analysis than nuclear DNA, as it allows for whole-genome analysis [90]. Nuclear DNA, in turn, requires locus-specific analysis and can generate information bottlenecks and lead to wrong clustering. An evaluation of cpDNA sequences in stone pine showed low levels of polymorphism [48,49], which is concurrent with other DNA analysis phylogenetic approaches. Specifically for stone pine, the low levels of polymorphism are transversal to all types of DNA that are usually used in taxonomic identification, which validates its status as an extremely resilient species with a homogenous and conservated genetic background.

### 7.7. Proteins and Enzymes

The proteins present in certain plant tissues or processes open a window to the knowledge of plant proteomes. Proteomes are not only useful in understanding genetic diversity but also how and when genes are expressed [72]. So far, the proteome of stone pine has been used to try to differentiate geographically separate populations [35,36] and to study pathogen [30,37] and environmental stressor resistance [54,55]. Akin to proteins, the enzymes (biocatalytic proteins) present in the plant’s life cycle can also provide insights into genetic diversity [91]. Stone pine’s enzymatic profiles have been used in phylogenetic [39] and population [38] studies and even for species identification [40,43]. Additionally, several studies on enzymes involved in evolution and environmental responses [28,41,53], seed physiology [42,45], and pathogen resistance [31,32,52] can be found for stone pine. Enzyme diversity is also a topic of interest [44,45,46], actively contributing to the knowledge of the stone pine proteome. It can be argued that evaluating the proteome is not enough to understand the full genetic components behind a species. This does not invalidate the use of protein profile analysis as a great complementary tool to understand the genetics behind stone pine’s features.

## 8. Conclusions

The full genome of stone pine is yet to be fully decoded, and its genetic information is understudied compared to other members of the Pinaceae family.

Studies on the stone pine transcriptome have identified differentially expressed genes during adventitious shoot bud formation and embryogenesis, indicating the involvement of multiple genes in these processes. The stone pine proteome reveals a variation and differentiation among populations and the dynamics of the needle proteome in response to fungal infection.

Microsatellites have been extensively studied in stone pine and are the most researched genetic markers for this species. Stone pine exhibits low levels of microsatellite polymorphism. The low genetic diversity observed in stone pine is unique among species with such a wide geographic distribution.

Fungal pathogens, including *H. annosum*, *H. irregulare*, and *F. circinatum*, have been studied in the context of stone pine–fungal pathogen interactions. Stone pine shows natural resistance to *F. circinatum*, and studying this plant–pathogen interaction can provide insights into resistance mechanisms and guide antifungal strategies. Pathogenesis-related proteins and genes associated with osmotic stress, oxidoreductive processes, and cell death are expressed in response to pinewood nematode infection.

Different pine species have distinct terpene, fatty acid, and long-chain alcohol profiles, providing a chemical fingerprint that allows for distinguishing stone pine from other pine species and subspecies.

Several morphophysiological aspects of stone pine, including cone and seed yield and growth traits, demonstrate phenotypic plasticity, making them useful markers for evaluating the species’ adaptability. Environmental factors, such as soil conditions and climate, have a more significant impact on stone pine’s growth parameters than population genetics.

### Challenges and Future Directions

Within the scope of this review, data analysis showed that most articles cite each other, with the exception of seven isolated articles. In fact, there are few publications available focusing on the genetics of *P. pinea*, which reveals a great gap in the literature, with the main ones being related to functional genes and genes related to the response to external factors. The way this species genome is regulated and how it modulates the plant’s phenotype remain understudied. The few works carried out so far have resulted in many important findings that can change the way we see this species. Its resistance to pathogens (fungi and nematodes) and high tolerance to drought make stone pine a model tree for climate change adaptation and reforestation efforts. This highly resilient tree proves to be extremely valuable in ever-changing ecosystems, where it can thrive as an added-value forest product, helping to boost the bioeconomy of forest regions.

## Figures and Tables

**Table 1:** Genes reported in literature for stone pine. The table contains the gene and its transcript, main functions, and the reference where it is mentioned. The references are organized chronologically.

Gene	Transcript	Function	References
AOX	Alternative oxidase	Response to increases in atmospheric CO_2_	[28]
PipiRR1	Type-A response regulator	Involved in cytokinin signal transduction pathway	[29]
ATTRX1	Arabidopsis thaliana thioredoxin H-type 1	Thiol–disulfide exchange intermediate	[30]
MAT2	Methionine adenosyltransferase 2	DNA and histone methylation	[30]
SAM2	S-adenosylmethionine synthase 2	Ethylene, nicotianamine, and polyamine biosynthetic pathways; provides the methyl group for protein and DNA methylation	[30]
PpnAPS	Pinus pinea α-pinene synthase	Synthesis of α-pinene (component of essential oils)	[31]
PAL	Phenylalanine ammonia-lyase	Phenylpropanoid metabolic network	[32]
CAD	Cinnamyl alcohol dehydrogenase	Lignin biosynthesis	[32]
XET	Xyloglucan endo-transglycosylase	Mechanical reinforcement of cell wall	[32]
CHI	Chitinase	Degradation of chitin in fungal cells	[32]
PAS1	FKBP-type peptidyl-prolyl cis-trans isomerase family protein	Determination of sensitivity to cytokinin for cell division	[33]
CLV1	Leucine-rich receptor-like protein	Promote tissue differentiation by suppressing the WUSCHEL gene (stem cell identity expression factor)	[33]
SNF2 domain	Putative helicase	Chromatin remodeling, trans-acting transcriptional regulators, and general transcription machinery	[33]
ATAF1	Arabidopsis Transcription Activator Factor-1	Meristem formation	[33]
ACBF	Polyadenylate-binding protein RBP47-like	Transcriptional regulation during shoot induction; petal development and xylogenesis	

**Table 2:** Microsatellites reported in literature for stone pine. The table contains the type of DNA from which the microsatellite was sequenced and the main results of its study. The references are organized chronologically.

Type of DNA	Results	References
Nucleus	No trans-specific microsatellite was polymorphic, de novo development of microsatellites seems mandatory.	[47]
Chloroplast and nucleus	Low levels of polymorphism	[48]
Chloroplast	Lack in polymorphism can be an advantage for adaptation	[49]
Chloroplast and nucleus	Low levels of polymorphism	[50]
Nucleus	Low levels of polymorphism	[19]

**Table 3:** Environmental stressors’ responses reported in literature for stone pine. The table contains the type of environmental stressor, the activated pathways, and the plants’ response. The references are organized chronologically.

Environmental Stressor	Pathway	Response	References
UV-B radiation	Expression of superoxide dismutase	Reduction in oxidative stress	[53]
Atmospheric CO_2_	Expression of AOX genes	Not yet determined	[28]
Water stress	Increased expression of glycosyltransferases, galactosidases, sugar transporters, dehydrins, and transcription factors	Higher metabolism, cell rescue, and intercellular transport	[54]
	Expression of K2-dehydrins	Accumulation of proteins in aerial parts of the plant to signal water retention	[55]

**Table 4:** Chemotypes (terpene profiles) reported in literature for stone pine. The references are organized chronologically.

Chemotype	Compounds	References
Chemotype D	Limonene, germacrene D, α-pinene, β-pinene	[56]
Not named	α-pinene, β-pinene, (-)-limonene	[57]
PPL1/PPL2/PPL3	β-pinene, terpinolene, α-pinene, 3-carene, sylvestrene, germacrene D, isocaryophyllene	[58]

**Table 5:** Fatty acid profiles reported in literature for stone pine. The compounds are organized by number of carbons and alphabetically.

Compounds (Fatty Acids)	N. Carbons	References
Myristic acid	14	[46]
Palmitic acid	16	[28,46,47]
Palmitoleic acid	16	[46]
14-methylhexadecanoic acid	17	[41]
cis-9-heptadecenoic acid	17	[46]
Heptadecanoic acid	17	[46]
cis-9,12-linoleic acid	18	[46,47]
cis-9,12,15-linolenic acid	18	[46]
cis-9-oleic acid	18	[46,47]
Linoleic acid	18	[41]
Oleic acid	18	[41]
Pinolenic acid	18	[41]
Stearic acid	18	[28,46,47]
Arachidic acid	20	[28,46]
cis-5,11,14-dihomo-γ-linolenic acid	20	[47]
cis-11,14-eicosadienoic acid	20	[46]
cis-11-gondoic acid	20	[46]
Sciadonic acid	20	[41]
Behenic acid	22	[41]

## References

[1] Fernandes P.M., Vega J.A., Jiménez E., Rigolot E. (2008). Fire Resistance of European Pines. For. Ecol. Manag..

[2] Fady B., Esposito E., Abulaila K., Aleksic J.M., Alia R., Alizoti P., Apostol E.N., Aravanopoulos P., Ballian D., Kharrat M.B.D. (2022). Forest Genetics Research in the Mediterranean Basin: Bibliometric Analysis, Knowledge Gaps, and Perspectives. Curr. For. Rep..

[3] Martínez F., Montero G. (2004). The *Pinus pinea* L. Woodlands along the Coast of South-Western Spain: Data for a New Geobotanical Interpretation. Plant Ecol..

[4] Zaibet L. (2016). Potentials of Non-Wood Forest Products for Value Chain Development, Value Addition and Development of Nwfp-Based Rural Microenterprises: Tunisia.

[5] Fady B., Fineschi S., Vendramin G.G. (2004). Technical Guidelines for Genetic Conservation and Use for Italian Stone Pine (Pinus pinea).

[6] Afonso A., Gonçalves A.C., Pereira D.G. (2020). *Pinus pinea* (L.) Nut and Kernel Productivity in Relation to Cone, Tree and Stand Characteristics. Agrofor. Syst..

[7] Awan H.U.M., Pettenella D. (2017). Pine Nuts: A Review of Recent Sanitary Conditions and Market Development. Forests.

[8] Mutke S., Calama R., González-Martínez S.C., Montero G., Gordo F.J., Bono D., Gil L. (2012). Mediterranean Stone Pine: Botany and Horticulture. Hortic. Rev..

[9] TechNavio Global Pine Nuts Market 2020–2024. https://www.researchandmarkets.com/reports/5180892/global-pine-nuts-market-2020-2024.

[10] International Nut & Dried Fruit Council Congress Presentations: Nut and Dried Fruit Production Prospects and Expert Sessions. https://inc.nutfruit.org/congress-presentations-nut-and-dried-fruit-production-prospects-and-expert-sessions/.

[11] Expert Market Pine Nuts Market Size, Type Analysis, Application Analysis, End-Use, Industry Analysis, Regional Outlook, Competitive Strategies And Forecasts, 2023–2032. https://www.marketexpertz.com/report/pine-nuts-market.

[12] Jaouadi W., Alsubeie M., Mechergui K., Naghmouchi S. (2021). Silviculture of *Pinus pinea* L. in North Africa and The Mediterranean Areas: Current Potentiality and Economic Value. J. Sustain. For..

[13] Mechergui K., Naghmouchi S., Altamimi A.S., Jaouadi W. (2021). Evaluation of Biomass, Carbon Storage Capability, Agroforestry of *Pinus pinea* L. and Management Practices to Increase Stocks: A Review. CERNE.

[14] Pereira S., Prieto A., Calama R., Diaz-Balteiro L. (2015). Optimal Management in *Pinus pinea* L. Stands Combining Silvicultural Schedules for Timber and Cone Production. Silva Fenn..

[15] Adelina N.M., Wang H., Zhang L., Yang K., Zhang L., Zhao Y. (2022). Evaluation of Roasting Conditions as an Attempt to Improve Bioactive Compounds and Antioxidant Activities of Pine Nut Shell and Skin. Waste Biomass Valoriz..

[16] Allegrini A., Salvaneschi P., Schirone B., Cianfaglione K., Di Michele A. (2022). Multipurpose Plant Species and Circular Economy: *Corylus Avellana* L. as a Study Case. Front. Biosci. Landmark.

[17] Lazaridou D.C., Michailidis A., Trigkas M. (2021). Exploring Environmental and Economic Costs and Benefits of a Forest-Based Circular Economy: A Literature Review. Forests.

[18] Sattout E., Faour G., Carrasquinho I., Correia A.C., Mutke S. (2017). Insights on the Value Chain and Management Practices of Stone Pine Forests in Lebanon. Mediterranean Pine Nuts from Forests and Plantations.

[19] Jaramillo-Correa J.P., Bagnoli F., Grivet D., Fady B., Aravanopoulos F.A., Vendramin G.G., González-Martínez S.C. (2020). Evolutionary Rate and Genetic Load in an Emblematic Mediterranean Tree Following an Ancient and Prolonged Population Collapse. Mol. Ecol..

[20] Akyol A., Örücü Ö.K. (2019). Investigation and Evaluation of Stone Pine (*Pinus pinea* L.) Current and Future Potential Distribution under Climate Change in Turkey. Cerne.

[21] Maggini F., Baldassini S. (1995). Ribosomal RNA Genes in the Genus Pinus. I. Caryologia.

[22] Krupkin A.B., Liston A., Strauss S.H. (1996). Phylogenetic Analysis of the Hard Pines (Pinus Subgenus Pinus, Pinaceae) from Chloroplast DNA Restriction Site Analysis. Am. J. Bot..

[23] Marrocco R., Gelati M.T., Magglnl F., Maggini F. (1996). Nucleotide Sequence of the Internal Transcribed Spacers and 5.8s Region of Ribosomal DNA in *Pinus pinea* L. DNA Seq..

[24] Gernandt D.S., Liston A. (1999). Internal Transcribed Spacer Region Evolution in Larix and Pseudotsuga (Pinaceae). Am. J. Bot..

[25] Evaristo I., Santos S., Tenreiro R., Costa R. (2008). Comparison of Genetic Structure Assessed by Amplified Fragment Length Polymorphism and Retrotransposon-Based Sequence-Specific Amplification Polymorphism for Portuguese Populations of *Pinus pinea* L. Silvae Genet..

[26] Georgolopoulos G., Parducci L., Drouzas A.D. (2016). A Short Phylogenetically Informative CpDNA Fragment for the Identification of Pinus Species. Biochem. Syst. Ecol..

[27] Ballin N.Z., Mikkelsen K. (2016). Polymerase Chain Reaction and Chemometrics Detected Several Pinus Species Including Pinus Armandii Involved in Pine Nut Syndrome. Food Control.

[28] Frederico A.M., Zavattieri M.A., Campos M.D., Cardoso H.G., McDonald A.E., Arnholdt-Schmitt B. (2009). The Gymnosperm *Pinus pinea* Contains Both AOX Gene Subfamilies, AOX1 and AOX2. Physiol. Plant..

[29] Cortizo M., Álvarez J.M., Rodríguez A., Fernández B., Ordás R.J. (2010). Cloning and Characterization of a Type-A Response Regulator Differentially Expressed during Adventitious Shoot Formation in *Pinus pinea* L. J. Plant Physiol..

[30] Franco A.R., Santos C., Roriz M., Rodrigues R., Lima M.R.M., Vasconcelos M.W. (2011). Study of Symptoms and Gene Expression in Four Pinus Species after Pinewood Nematode Infection. Plant Genet. Resour..

[31] Trindade H., Sena I., Figueiredo A.C. (2016). Characterization of α-Pinene Synthase Gene in Pinus Pinaster and *P. pinea* in Vitro Cultures and Differential Gene Expression Following Bursaphelenchus Xylophilus Inoculation. Acta Physiol. Plant..

[32] Pepori A.L., Michelozzi M., Santini A., Cencetti G., Bonello P., Gonthier P., Sebastiani F., Luchi N. (2018). Comparative Transcriptional and Metabolic Responses of *Pinus pinea* to a Native and a Non-Native Heterobasidion Species. Tree Physiol..

[33] Alonso P., Cortizo M., Cantón F.R., Fernández B., Rodríguez A., Centeno M.L., Cánovas F.M., Ordás R.J. (2007). Identification of Genes Differentially Expressed during Adventitious Shoot Induction in *Pinus pinea* Cotyledons by Subtractive Hybridization and Quantitative PCR. Tree Physiol..

[34] Trontin J.F., Klimaszewska K., Morel A., Hargreaves C., Lelu-Walter M.A. (2016). Molecular Aspects of Conifer Zygotic and Somatic Embryo Development: A Review of Genome-Wide Approaches and Recent Insights. Methods in Molecular Biology.

[35] Alvarez J.B., Toledo M.J., Abellanas B., Martín L.M. (2004). Use of Megagametophyte Storage Proteins as Markers of the Genetic in Stone Pine (*Pinus pinea* L.) in Andalucia, Spain. Genet. Resour. Crop Evol..

[36] Loewe V., Navarro-Cerrillo R.M., Sánchez Lucas R., Ruiz Gómez F.J., Jorrín-Novo J. (2018). Variability Studies of Allochthonous Stone Pine (*Pinus pinea* L.) Plantations in Chile through Nut Protein Profiling. J. Proteom..

[37] Amaral J., Lamelas L., Valledor L., Castillejo M.Á., Alves A., Pinto G. (2021). Comparative Proteomics of Pinus–Fusarium Circinatum Interactions Reveal Metabolic Clues to Biotic Stress Resistance. Physiol. Plant..

[38] Fallour D., Lefevre F. (1997). Study on Isozyme Variation in *Pinus pinea* L.: Evidence for Low Polymorphism. Silvae Genet..

[39] Gad M.A., Mohamed S.Y. (2012). Phylogenetic Evaluation Of Some Pinus Species From Different Genetic Using Protein, Isozymes, RAPD And ISSR Analyses. J. Am. Sci..

[40] Stermitz F.R., Tawara J.N., Boeckl M., Pomeroy M., Foderaro T.A., Todd F.G. (1994). Piperidine alkaloid content of picea (spruce) and pinus (pine). Phytochemistry.

[41] Wolff R.L., Comps B., Marpeau A.M., Deluc L.G. (1997). Taxonomy of Pinus species based on the seed oil fatty acid compositions. Trees.

[42] Tommasi F., Paciolla C., Arrigoni O. (1999). The Ascorbate System in Recalcitrant and Orthodox Seeds. Physiol. Plant..

[43] González-Andrés F., Pita J.M., Ortiz J.M. (1999). Identification of Iberian and Canarian Species of the Genus Pinus with Four Isoenzyme Systems. Biochem. Syst. Ecol..

[44] Ranaldi F., Vanni P., Giachetti E. (2000). Multisite Inhibition of *Pinus pinea* Isocitrate Lyase by Phosphate. Plant Physiol..

[45] Faraoni P., Sereni E., Gnerucci A., Cialdai F., Monici M., Ranaldi F. (2019). Glyoxylate Cycle Activity in *Pinus pinea* Seeds during Germination in Altered Gravity Conditions. Plant Physiol. Biochem..

[46] Hu Y., Zhang C., Zou L., Zheng Z., Ouyang J. (2022). Efficient Biosynthesis of Pinosylvin from Lignin-Derived Cinnamic Acid by Metabolic Engineering of Escherichia Coli. Biotechnol. Biofuels Bioprod..

[47] González-Martínez S.C., Robledo-Arnuncio J.J., Collada C., Díaz A., Williams C.G., Alía R., Cervera M.T. (2004). Cross-Amplification and Sequence Variation of Microsatellite Loci in Eurasian Hard Pines. Theor. Appl. Genet..

[48] Vendramin G.G., Fady B., González-Martínez S.C., Hu F.S., Scotti I., Sebastiani F., Soto Á., Petit R.J. (2008). Genetically Depauperate but Widespread: The Case of an Emblematic Mediterranean Pine. Evolution.

[49] Soto A., Robledo-Arnuncio J.J., González-Martínez S.C., Smouse P.E., Alí R. (2010). Climatic Niche and Neutral Genetic Diversity of the Six Iberian Pine Species: A Retrospective and Prospective View. Mol. Ecol..

[50] Pinzauti F., Sebastiani F., Budde K.B., Fady B., González-Martínez S.C., Vendramin G.G. (2012). Nuclear Microsatellites for *Pinus pinea* (Pinaceae), a Genetically Depauperate Tree, Andtheir Transferability to *P. Halepensis*. Am. J. Bot..

[51] Zamora-Ballesteros C., Pinto G., Amaral J., Valledor L., Alves A., Diez J.J., Martín-García J. (2021). Dual RNA-Sequencing Analysis of Resistant (*Pinus pinea*) and Susceptible (Pinus Radiata) Hosts during Fusarium Circinatum Challenge. Int. J. Mol. Sci..

[52] Santos C.S., Pinheiro M., Silva A.I., Egas C., Vasconcelos M.W. (2012). Searching for Resistance Genes to Bursaphelenchus Xylophilus Using High Throughput Screening. BMC Genom..

[53] Petropoulou Y., Kyparissis A., Nikolopoulos D., Manetas Petropoulou Kyparissis Y.Y. (1995). Enhanced UV-B radiation alleviates the adverse effects of summer drought in two Mediterranean pines under field conditions. Physiol. Plant..

[54] Perdiguero P., Barbero M.d.C., Cervera M.T., Collada C., Soto Á. (2013). Molecular Response to Water Stress in Two Contrasting Mediterranean Pines (Pinuspinaster and *Pinus pinea*). Plant Physiol. Biochem..

[55] Perdiguero P., Soto Á., Collada C. (2015). Comparative Analysis of *Pinus pinea* and Pinus Pinaster Dehydrins under Drought Stress. Tree Genet. Genomes.

[56] Roussis V., Petrakis P.V., Ortiz A., Mazomenos B.E. (1995). Volatile Constituents of Needles of Five Pinus Species Grown in Greece. Phytochemistry.

[57] da Silva M.D.R.G., Mateus E.P., Munhá J., Drazyk A., Farrall M.H., Paiva M.R., Neves H.J.C.D. (2001). Differentiation of Ten Pine Species from Central Portugal by Monoterpene Enantiomer-Selective Composition Analysis Using Multidimensional Gas Chromatography. Chromatographia.

[58] Gad H., Al-Sayed E., Ayoub I. (2021). Phytochemical Discrimination of Pinus Species Based on GC–MS and ATR-IR Analyses and Their Impact on Helicobacter Pylori. Phytochem. Anal..

[59] Nasri N., Khaldi A., Hammami M., Triki S. (2005). Fatty Acid Composition of Two Tunisian Pine Seed Oils. Biotechnol. Prog..

[60] Nasri N., Khaldi A., Fady B., Triki S. (2005). Fatty Acids from Seeds of *Pinus pinea* L.: Composition and Population Profiling. Phytochemistry.

[61] João Gaspar M., Nunes J., Rodrigues M., Ferreira L. (2023). Chemotaxonomic Differentiation of Pinus Species Based on N-Alkane and Long-Chain Alcohol Profiles of Needle Cuticular Waxes. Chem. Biodivers..

[62] Pardos M., Calama R. (2018). Responses of *Pinus pinea* Seedlings to Moderate Drought and Shade: Is the Provenance a Differential Factor?. Photosynthetica.

[63] Andivia E., Zuccarini P., Grau B., de Herralde F., Villar-Salvador P., Savé R. (2019). Rooting Big and Deep Rapidly: The Ecological Roots of Pine Species Distribution in Southern Europe. Trees Struct. Funct..

[64] Férriz M., Martin-Benito D., Fernández-de-Simón M.B., Conde M., García-Cervigón A.I., Aranda I., Gea-Izquierdo G. (2023). Functional Phenotypic Plasticity Mediated by Water Stress and [CO2] Explains Differences in Drought Tolerance of Two Phylogenetically Close Conifers. Tree Physiol..

[65] Bogdziewicz M., Szymkowiak J., Tanentzap A.J., Calama R., Marino S., Steele M.A., Seget B., Piechnik Ł., Żywiec M. (2021). Seed Predation Selects for Reproductive Variability and Synchrony in Perennial Plants. New Phytol..

[66] Mutke S., Arias B., Sauce S., Sánchez L. (2003). Evaluación de La Producción Individual de Piña En Un Banco Clonal de Pino Piñonero (*Pinus pinea* L.) En Madrid. Investig. Agrar. Sist. Recur. For..

[67] Carrasquinho I., Gonçalves E. (2013). Genetic Variability among *Pinus pinea* L. Provenances for Survival and Growth Traits in Portugal. Tree Genet. Genomes.

[68] Mutke S., Gordo J., Khouja M.L., Fady B. (2013). Mediterranean Stone Pine for Agroforestry Low Genetic and High Environmental Diversity at Adaptive Traits in *Pinus pinea* from Provenance Tests in France and Spain. Options Méditerranéennes A.

[69] Loewe-Muñoz V., Balzarini M., Del Río R., Delard C. (2019). Effects of Stone Pine (*Pinus pinea* L.) Plantation Spacing on Initial Growth and Conelet Entry into Production. New For..

[70] Brunori A., D’amato F. (1967). The DNA Content of Nuclei in the Embryo of Dry Seeds of *Pinus pinea* and Lactuca Sativa. Caryologia.

[71] David H., De Boucaud M.-T., Gaultier J.-M., David A. (1986). Sustained Division of Protoplast-Derived Cells from Primary Leaves of Pinus Pinaster, Factors Affecting Growth and Change in Nuclear DNA Content. Tree Physiol..

[72] Bouchez D., Höfte H. (1998). Functional Genomics in Plants. Plant Physiol..

[73] Kovach A., Wegrzyn J.L., Parra G., Holt C., Bruening G.E., Loopstra C.A., Hartigan J., Yandell M., Langley C.H., Korf I. (2010). The Pinus Taeda Genome Is Characterized by Diverse and Highly Diverged Repetitive Sequences. BMC Genom..

[74] Fernández-Pozo N., Canales J., Guerrero-Fernández D., Villalobos D.P., Díaz-Moreno S.M., Bautista R., Flores-Monterroso A., Guevara M.Á., Perdiguero P., Collada C. (2011). EuroPineDB: A High-Coverage Web Database for Maritime Pine Transcriptome. BMC Genom..

[75] Parchman T.L., Gompert Z., Mudge J., Schilkey F.D., Benkman C.W., Buerkle C.A. (2012). Genome-Wide Association Genetics of an Adaptive Trait in Lodgepole Pine. Mol. Ecol..

[76] Baison J., Vidalis A., Zhou L., Chen Z.Q., Li Z., Sillanpää M.J., Bernhardsson C., Scofield D., Forsberg N., Grahn T. (2019). Genome-Wide Association Study Identified Novel Candidate Loci Affecting Wood Formation in Norway Spruce. Plant J..

[77] Mutke S., Gil L. (2000). The Stone Pine (*Pinus pinea* L.) Breeding Programme in Castile-Leon (Central Spain). NUCIS Newsl..

[78] Loewe Muñoz V., Balzarini M., Delard Rodríguez C., Álvarez Contreras A., Navarro-Cerrillo R.M. (2017). Growth of Stone Pine (*Pinus pinea* L.) European Provenances in Central Chile. IForest.

[79] Bartholomé J., Bink M.C., Van Heerwaarden J., Chancerel E., Boury C., Lesur I., Isik F., Bouffier L., Plomion C. (2016). Linkage and Association Mapping for Two Major Traits Used in the Maritime Pine Breeding Program: Height Growth and Stem Straightness. PLoS ONE.

[80] Liu J.J., Schoettle A.W., Sniezko R.A., Yao F., Zamany A., Williams H., Rancourt B. (2019). Limber Pine (Pinus Flexilis James) Genetic Map Constructed by Exome-Seq Provides Insight into the Evolution of Disease Resistance and a Genomic Resource for Genomics-Based Breeding. Plant J..

[81] McLean D., Apiolaza L., Paget M., Klápště J. (2023). Simulating Deployment of Genetic Gain in a Radiata Pine Breeding Program with Genomic Selection. Tree Genet. Genomes.

[82] Dzialuk A., Chybicki I., Gout R., Mączka T., Fleischer P., Konrad H., Curtu A.L., Sofletea N., Valadon A. (2014). No Reduction in Genetic Diversity of Swiss Stone Pine (*Pinus cembra* L.) in Tatra Mountains despite High Fragmentation and Small Population Size. Conserv. Genet..

[83] Przybylski P., Tereba A., Meger J., Szyp-Borowska I., Tyburski Ł. (2022). Conservation of Genetic Diversity of Scots Pine (*Pinus sylvestris* L.) in a Central European National Park Based on CpDNA Studies. Diversity.

[84] Yu Y., Aitken S.N., Rieseberg L.H., Wang T. (2022). Using Landscape Genomics to Delineate Seed and Breeding Zones for Lodgepole Pine. New Phytol..

[85] Kress W.J., Erickson D.L. (2012). DNA Barcodes: Methods and Protocols. Methods Mol. Biol..

[86] Neumann P., Novák P., Hoštáková N., MacAs J. (2019). Systematic Survey of Plant LTR-Retrotransposons Elucidates Phylogenetic Relationships of Their Polyprotein Domains and Provides a Reference for Element Classification. Mob. DNA.

[87] Zimmer E.A., Wen J. (2015). Using Nuclear Gene Data for Plant Phylogenetics: Progress and Prospects II. Next-Gen Approaches. J. Syst. Evol..

[88] Kalia R.K., Rai M.K., Kalia S., Singh R., Dhawan A.K. (2011). Microsatellite Markers: An Overview of the Recent Progress in Plants. Euphytica.

[89] Knoop V. (2004). The Mitochondrial DNA of Land Plants: Peculiarities in Phylogenetic Perspective. Curr. Genet..

[90] Martin W., Deusch O., Stawski N., Grünheit N., Goremykin V. (2005). Chloroplast Genome Phylogenetics: Why We Need Independent Approaches to Plant Molecular Evolution. Trends Plant Sci..

[91] Bohlmann J., Meyer-Gauen G., Croteau R. (1998). Plant Terpenoid Synthases: Molecular Biology and Phylogenetic Analysis. Proc. Natl. Acad. Sci. USA.

